# AS-LAMP: A New and Alternative Method for Genotyping

**Published:** 2020

**Authors:** Pooria Gill, Arash Hadian Amree

**Affiliations:** 1. Department of Medical Nanotechnology, Faculty of Advanced Technologies in Medicine, Mazandaran University of Medical Sciences, Sari, Iran; 2. Student Research Committee, Thalassemia Research Center, Hemoglobinopathy Institute, Mazandaran University of Medical Sciences, Sari, Iran

**Keywords:** AS-LAMP, Genotyping, LAMP, Single nucleotide polymorphisms

## Abstract

In recent decades, different methods have been introduced for the genotyping of Single Nucleotide Polymorphisms (SNPs) and mutations in nucleic acid sequences. These methods have several applications ranging from agriculture to medicine. The Loop-mediated isothermal amplification (LAMP) method was first introduced by Notomi *et al*. Since then, different methods derived from LAMP have been extensively applied in detecting pathogens. The LAMP method is an isothermal technique that amplifies the target DNA segment using four different primers that have been uniquely designed for recognizing six distinct zones on the objective gene; the process of reaction continues at a constant temperature *via* a strand displacement reaction. Amplifying and detecting the targeted zone can be accomplished in one stage. Although the LAMP method is mostly used for pathogen detection, several studies have used this method for genotyping. The present article reviewed various studies that used the LAMP method for SNP detection. The outcomes indicated that the LAMP technique could be a reliable and alternative technique for genotyping. Further studies are recommended to use this approach for genotyping.

## Introduction

Finding the changes that occur in nucleic acid sequences is an important issue in various fields ranging from medicine to agriculture. Because the genomes of all organisms consist of a huge amount of DNA, it is very difficult to directly detect Single Nucleotide Polymorphisms (SNPs) or nucleotide changes in a genome. Currently, nucleic acid amplification methods are extensively utilized in many labs for identifying variations in previously well-characterized genomic regions 1. Since its innovation in 1993, the Polymerase Chain Reaction (PCR) has been the basis for most widespread techniques for applying nucleic acid amplification 2–6. Although PCR-based methods are cost-effective techniques that are very robust for nucleic acid amplification, they cannot be performed in every lab. In addition, the methods require a thermocycling system, which limits their application in the field 7,8.

In the past years, numerous nucleic amplification approaches have been introduced that do not need a thermocycling system. Isothermal techniques to amplify nucleic acids include Loop-mediated isothermal Amplification (LAMP), Nucleic Acid Sequence-Based Amplification (NASBA), Helicase-Dependent Amplification (HDA), Rolling Circle Amplification (RCA), Multiple Displacement Amplification (MDA), and Re-combinase Polymerase Amplification (RPA); these new approaches can easily be used at a constant temperature on a simple heater block 9–16.

## LAMP Method

**Figure 1.**
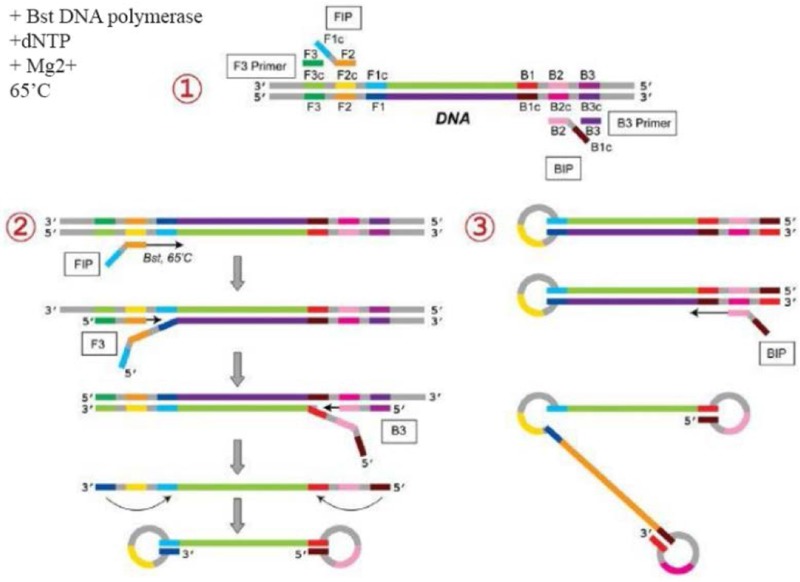
Schematic representation of the Loop-mediated isothermal Amplification for (LAMP). In the first step, the 435 designed primers bind to the complementary sequences. Then, the dumbbell-like DNA form is generated. Next, 436 in the cycling amplification step, DNAs of this form are generated continuously. The elongation reactions are 437 started from the products of the cycling amplification step, generating various sizes of the products.

The LAMP method was first introduced by Notomi *et al* in 2000 17. Since then, different methods derived from LAMP have been widely used for pathogen detection 18–21. The LAMP method uses four sets of primers that are specially aimed to identify six distinct zones on the objective gene. These sets of primers are the outer primers (F3 and B3) and the inner primers. The forward inner primer (FIP) consisting of the F2 zone at the 3′ end with the sequence similar to the F1c zone at the 5′ end, is complementary to the F2c zone, while the Backward Inner Primer (BIP) consisting of the B2 zone at the 3′ end with the sequence similar to the B1c region at the 5′ end, is complementary to the B2c zone. The two primers are complementary to the downstream zone of the opposite strand in the objective (F1 and B1) 17. In addition to the specific primers, the LAMP reaction requires Bst polymerase, deoxynucleotide triphosphates (dNTPs), magnesium sulfate, betaine, and buffer for enzyme 18. The products of amplification, which have a stem–loop DNA structure, contain numerous inverted repeats of the objective region and cauliflower-like structures with multiple loops 22 (Figure 1). The LAMP method can quickly amplify a large number of DNA usually at 60*°C*, which leads to the production of pyrophosphate and the production of a white sediment, which is magnesium pyrophosphate 23. Although observing the white precipitate can indicate that the nucleic acid was amplified, LAMP products can also be identified using gel electrophoresis, real-time turbidimetry, fluorescent probes, and SYBR green dye 23–25. The LAMP method yields a considerable amount of amplicons in a short time (around 60 *min*), which is 103 times higher than the amount produced with simple PCR 26. Because the LAMP method uses four primer sets that are specific to the objective regions, it has rapid amplification, a higher amount of amplification products, and smaller detection limits compared with PCR-based methods 26.

Unlike PCR, which needs thermocycling system, the LAMP reaction can be performed at a fixed temperature and does not need a thermocycling system. Hence, LAMP is more beneficial for Point-Of-Care Testing (POCT) than PCR 21. It should be mentioned that the LAMP procedure also has some limitations, such as the complications involved in designing primers and the difficulties in using LAMP in multiplex reactions.

### SNP detection using the LAMP method

Since its introduction, the LAMP technique has been applied for detecting infections in which the amplification of the pathogen genome is required. The LAMP method has rarely been used for genotyping and detecting point mutations. LAMP is a sensitive technique that can quickly amplify the target DNA in an isothermal condition. Thus, designing new LAMP methods that could be used for genotyping is very important because the introduced methods could be a suitable alternative to other genotyping techniques. Moreover, the LAMP technique is a very suitable, easy to use, and cost-effective approach for POCT or on-site testing.

The current article was aimed to study the existing evidence for genotype detection using the LAMP technique as an isothermal approach. In addition, the advantages and properties of the LAMP technique were also discussed.

#### AS-LAMP

In the Allele-Specific (AS)–LAMP method, two sets of LAMP primers are provided for distinguishing between two diverse nucleotides in the sequence corresponding to a target gene. Specific primers, two primers of BIP or FIP, are planned with the mutation point at the 3′ end corresponding to the B2 primer (5′ end corresponding to the BIP primer) and an extra mismatched nucleotide (lower case letter) to enhance the specificity to every target nucleotide site. In the other two primer sets, the primers of F3, B3, and FIP or BIP, are the same. For each sample, two LAMP reactions are applied with each set of primers.

Various studies used the AS-LAMP method for genotyping. For example, the method was used for the identification of the West African kdr (kdr-w; L1014F) 27 and G119S ace-1R mutations 28 in field-collected *Anopheles gambiae*.

Yongkiettrakul *et al* also used an SNP-LAMP assay for detecting the N51I mutation on the *dhfr* gene, which is related to pyrimethamine resistance in *Plasmodium falciparum* 29. The SNP-LAMP method show-ed 100% specificity 29.

Ikeda *et al* designed an Amplification-Refractory Mutation System (ARMS)-LAMP approach to detect an L858R mutation of the Epidermal Growth Factor Receptor (EGFR) 30. They used an in situ LAMP reaction on paraffin-embedded tissues. The FIP and BIP primers were labeled with Fluorescein Isothiocyanate (FITC). The comparison between outcomes of the in situ LAMP reaction with the results of PCR-Restriction Fragment Length Polymorphism (RFLP) showed that while the PCR-RFLP method could detect the mutation in 12 of 26 patients, the in situ LAMP technique detected the mutation in 15 patients 30.

Nakamura *et al* used the LAMP method combined with an electrochemical DNA chip to simultaneously identify the six polymorphisms related with Rheumatoid Arthritis (RA), including *N-acetyltransferase2* (*N-AT2*) gene polymorphisms T341C, G590A, and G857-A, *Methylenetetrahydrofolate reductase* (*MTHFR*) gene polymorphisms C677T and A1298C, and *Serum Amyloid A1* (*SAA1*) gene promoter polymorphism −13 C>T 31. In the study, 31 samples were genotyped using the combined LAMP technique and the outcomes were found to be in a good agreement with the results from the PCR-RFLP 31.

Tamura *et al* developed a LAMP technique for detecting the N526K ftsI mutation corresponding to β-Lactamase-Negative Ampicillin-Resistant (BLNAR) *Haemophilus influenzae* (*H. influenzae*) 32. They combined the LAMP method with the ARMS approach to exactly identify a diverse single nucleotide in the objective sequence. The method could perform a species–specific identification of a nucleotide (1578T) in the *ftsI* gene of *H. influenzae* with no amplification of the point mutations (T1578G/A) in the BLNAR strains. The limit of detection was obtained (10.0 *pg* of genomic DNA per reaction) 32.

Duan *et al* developed a fast and effective approach with great specificity on the basis of LAMP for detecting the point mutation at codon 200 (TTC&x2192;TAC, F200Y) of the *β2-tubulin* gene, which shows resistance to benzimidazole fungicide in *Fusarium asiaticum* (*F. asiaticum*) 33. A comparison was performed between the outcomes of LAMP test and the outcomes of PCR test. The researchers found that the LAMP test could effectively identify the F200Y mutant genotype in carbendazim-resistant separates of *F. asiaticum* in agricultural crops 33. Lin *et al* used the AS-LAMP in order to distinguish between wild-type and vaccine strains corresponding to Mink enteritis virus 34.

Kwong *et al* applied SNP genotyping-LAMP method for identification of CYP2C19*2 and CYP2C19*3 SNPs on *CYP2C19* gene on 100 samples and the results were compared with the results of real-time PCR melting curve analysis. SYBR Safe DNA Gel Stain (a DNA intercalating dye) was added directly to the reaction tube for the visualizing of LAMP products. This dye would turn green under ambient light if the targeted LAMP products were amplified. It would remain orange if the products were not amplified. The real-time PCR melting curve analysis and SNP genotyping-LAMP assay had concordant results and both methods could successfully detect CYP2C19*2 and CYPC19*3 SNPs. Besides, they were able to differentiate heterozygous CYP2C19*2 variants from homozygous ones 35.

### LAMP-RFLP

A common method for genotyping in molecular biology involves the digestion of specific sequences using restriction enzymes. RFLP on the PCR product is one of the most common methods used for detecting SNPs in a genome 36–38. LAMP products can also be digested with specific restriction enzymes in order to identify the SNPs.

Shao *et al* developed a multiplex LAMP-RFLP (mLAMP-RFLP) for detecting Salmonella strains and Shigella strains in milk simultaneously 39. They used two sets of LAMP primers to definitely aim ipaH of Shigella spp and invA of Salmonella spp.

Yoshida *et al* developed a Reverse Transcription (RT)-LAMP technique mixed with RFLP to distinguish the Hoshino vaccine strain of the mumps virus from circulating wild strains 40. First, the Hemagglutinin Neuraminidase (HN) area corresponding to the virus was amplified using AMV reverse transcriptase and Bst DNA polymerase. In the HN area, the strain of vaccine has a particular restriction enzyme site of ScaI. The sensitive and differential method proved to be effective for the laboratory surveillance corresponding to vaccine-adverse events 40.

### LAMP method combined with an electrochemical oligonucleotide chip

In order to determine the copies of *CYP2D6* gene correctly, Nakamura *et al* planned a primer set that co-amplified the *CYP2D6* gene, but not the CYP2D6*36 and the *CYP2D8P* gene 41. The copies of *CYP2D6* gene were determined through comparison of the quantity of the amplified products of the *CYP2D6* gene with the *CYP2D8P* gene. For confirmation of the amplification product specificity, the digestion of products was performed with NcoI and Hpy99 restriction enzymes. The amplified products were hybridized on a chip containing probes that were complementary to the *CYP2D6* and *CYP2D8P* genes. The copies of CYP2D6 gene were in agreement with earlier tests. The introduced method lasted just for 1.5 *hr* 41.

### Exo-proofreading LAMP

Kuzuhara *et al* developed a SNP detecting version of LAMP combined with the 3′–5′ exonuclease proofreading activity corresponding to DNA polymerase called PF LAMP 42. A primer was designed so that its 3′ terminus was aligned at the polymorphic base of the DNA target and labeled with a detectable fluorescence tag on the 3′ nucleotide base. When the 3′ end corresponding to the primer was complementary to the objective, the labeled nucleotide remained and was combined with the extension product. When the primer did not match with the template, the proofreading activity of the polymerase removed the labeled nucleotide and no tag was combined with the extension product. The detection of the genotype was accomplished using fluorescence polarization with no extra cleanup. Using this method, the researchers could successfully detect the G1951A SNP in the human *Aldehyde Dehydrogenase 2* (*ALDH2*) gene 42.

### PNA-LNA mediated LAMP

**Figure 2.**
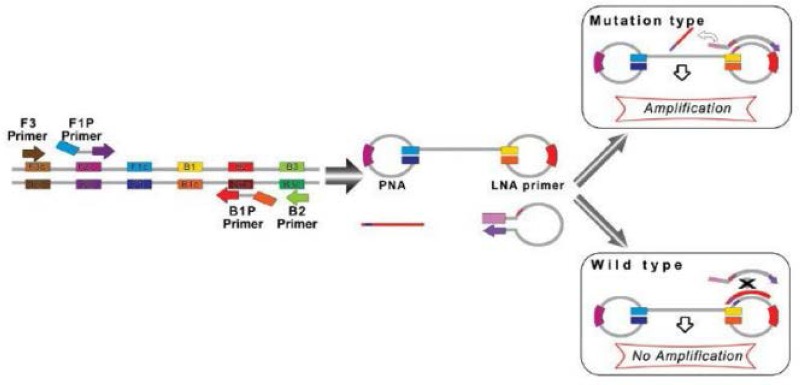
A schematic picture of PNA-LNA mediated LAMP. When the target is wild-type sequence, the 450 clamping PNA probe forms a stable duplex with the dumbbell structure, and interferes with the 451 annealing and elongation of the LNA primer. When the target DNA has mutated sequence, the 452 clamping PNA probe does not anneal with the DNA because of the single-base mismatch, and the 453 elongation reaction proceeds.

Itogana *et al* designed a Peptide Nucleic Acid–Locked Nucleic Acid (PNA-LNA) mediated LAMP method for detecting a KRAS mutation 43. In the PNA-LNA mediated LAMP method, a specific clamping PNA probe for the wild type nucleotide and extra LNA primers corresponding to the mutant type nucleotide were planned for the looped area corresponding to the main products of LAMP. The LAMP reactions were carried out under isothermal conditions at temperature of 65*°C* using a strand-displacement DNA polymerase. In the method, the FIP and B3 primers anneal and extend on the target DNA and the freshly prepared DNA chains are moved through extending F3 and B3, respectively. In the wild type samples, the PNA probe generates moved products with stem loop structures that prevent the annealing and extending the LNA primer. In the case of mutation of the objective gene, the PNA cannot form a clamp with the displaced DNA because of a single-base mismatch; as a result, the LNA primer attaches and anneals to the objective area and subsequently continues extending (Figure 2). The amplified product real-time PCR equipment was detected by agarose gel electrophoresis with the naked eye. A direct sequencing test and PNA-clamping PCR were compared with the PNA-LNA mediated LAMP technique in terms of the Limit of Detection (LOD) of the KRAS point mutation. Detection of mutant alleles could be reproducibly performed with a mutant-to-wild type ratio of 30% with direct sequencing and a ratio of 1% with PNA-clamping PCR, while the detection of mutant alleles could be performed over 50 *min* in specimens diluted to a mutant-to-wild type ratio of 0.1% using the LAMP method. Cao *et al* developed PNA-LNA clamping LAMP for the quick identification of CALR type 1 (CALR-1) and type 2 (CALR-2) mutations in Philadelphia chromosome-negative MPN subjects 44.

### Invasive reaction combined with oligonucleotide probe-modified gold nanoparticles

**Figure 3.**
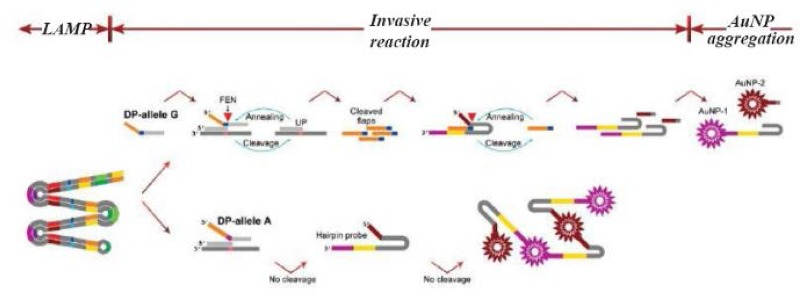
A schematic image of AS-LAMP coupled with invasive reaction and hybridization-induced AuNP 467 aggregation. The designed method contains three steps: LAMP, invasive reaction and AuNP aggregation. 468 LAMP amplifies the targeted sequence at first. Afu flap endonuclease specifically differentiate the invasive 469 structure formed by an upstream probe (UP), a downstream probe (DP), and a targeted sequence in the 470 amplicon, yielding the flaps to initiate the cleavage of hairpin probes. If the targeted allele is present, hairpin 471 probes is cleaved into two fragments, giving red color of the tube. While, if the targeted allele is absent, 472 cleavage of the hairpin probes does not occur, leading to AuNPs aggregation-induced precipitation.

Lu *et al* introduced a new technique derived from the LAMP method for SNP detection 45. The introduced method had a complicated structure. First, multiple fragments comprising the relevant SNPs were amplified using a set of three pairs of specific primers. Then the targeted base in the amplicon was recognized using two allele specific probes including an Upstream Probe (UP) and a Downstream Probe (DP). It should be mentioned that there were two designs of DP probes, one for a normal allele and the other for a mutated allele. Next, a subsequent invasive reaction using Afu Flap Endonuclease (FEN) was performed. In the presence of the target allele, the DP allele forms an overlapping structure that is identified and cleaved by FEN; the FEN cleaves the 5′ end of the DP probe. A hairpin should also be designed to capture the cleaved DP probes; the hairpin itself is cleaved by FEN in the presence of the cleaved DP probes. A pair of ends of the hairpin probe is designed for capturing gold nanoparticles (AuNPs) coated probes. Because of different sequences of these two ends, two types of AuNPs coated in two different probes should be applied. If the hairpin probe is not cleaved, the AuNPs on the probes captured by the hairpin probes cause the aggregation of AuNPs, which forms a precipitate in the test tubes. When the hairpin is cleaved because of the presence of the target sequence, the free AuNPs cause the solution to turn red (Figure 3). The researchers used naked-eye detection to identify the SNPs in the isothermal condition. Due to the sensitivity of the allele-specific probe to detect one base difference, they argued that the mLAMP method can be used to produce amplicon mixes for several SNP detections.

Lu *et al* then designed a mLAMP protocol for genotyping three diverse SNPs (CYP2C19*2, CYP2C19*3 and MDR1-C3435T), which are important in guiding the dose of clopidogrel 45. In fact, they used the 3-plex LAMP for objective amplification and the subsequent individual invader reaction for discrimination of every allele in the objective area. They indicated that 100 copies of genomic DNA are sufficient for giving a correct typing outcome for all three SNPs. Comparing the outcomes of the protocol with the outcomes of the pyrosequencing method on clinical samples showed identical typing results.

## Conclusion

In this review, different studies that used the LAMP method for genotyping and SNPs detection were analyzed. These studies combined the LAMP method with various technologies in order to produce a technique that was suitable for genotyping. Although the LAMP technique has mainly been applied to detect the genomes of infectious agents without the determination of the SNPs, the reviewed studies showed that the LAMP method could also be a good approach for the genotyping of different SNPs. Unlike the conventional LAMP method which is widely used for pathogen detection, the AS-LAMP is a newly introduced method and further studies are required to make it popular for researchers.

Because the LAMP method is a sharp, fast, and robust technique, it is also suitable for use in POC situations. Thus, the LAMP method combined with other approaches for genotyping is highly recommended for POC testing.
